# Lidocaine injections and neck corset wearing improve dropped head syndrome in Parkinson's disease and related disorders

**DOI:** 10.1016/j.prdoa.2019.10.003

**Published:** 2019-10-26

**Authors:** Yohei Mukai, Yoshihiko Furusawa, Yuko Morimoto, Yuka Hama, Tomoya Kawazoe, Yuji Saitoh, Takashi Sakamoto, Yuji Takahashi, Miho Murata

**Affiliations:** Department of Neurology, National Center Hospital, Parkinson's Disease & Movement Disorders Center, National Center of Neurology and Psychiatry, Japan

**Keywords:** Dropped head, Parkinson's disease, Muscle afferent block, Lidocaine, Scalene muscle

## Abstract

**Background:**

Patients with Parkinson's disease and related disorders (PDRD) may exhibit dropped head syndrome (DHS), which does not yet have an effective treatment.

**Objectives:**

To evaluate the effect of combining lidocaine injection into the bilateral scalene muscles and neck corset wearing on dropped head syndrome.

**Methods:**

We performed needle electromyography assessments of the scalene, sternocleidomastoid (SCM), levator scapulae, splenius capitis, and trapezius muscles. Patients received 2.5–5 ml injections of 1% lidocaine into both sides of the scalene muscles for 4/5 consecutive days and were instructed to wear a neck corset. We measured the neck flexion angle, which formed between the horizontal line and the straight line passing through the ear canal and orbital fossa, before (baseline) and after (Day 8 and Day 90) the intervention.

**Results:**

Seven males and eight females (mean age, 68.9 years; range 56 to 85 years) who had PDRD with dropped head syndrome were enrolled in this study. Needle electromyography examination revealed abnormal discharge of the scalene muscles in all patients when the neck position was corrected; however, some patients did not show abnormal discharge of the SCM muscle. At Day 8, we observed an improvement of the neck flexion angle in 13 of the 15 patients, from an average of 27.7° ± 13.9° to 11.7 ± 14.6°. At Day 90, the average neck flexion angle was 15.3° ± 17.2°.

**Conclusions:**

Combining lidocaine injection into the scalene muscles and neck corset wearing is an effective treatment regimen for DHS in patients with PDRD.

## 1 Introduction

Dropped head syndrome (DHS) refers to the state where the head is bent toward the trunk when in a sitting or standing position, and was once was described as floppy head syndrome [1]. The pathomechanism of DHS has been considered to involve excess contraction of the neck flexor muscles or weakness of the neck extensor muscles [2]. In general, the former mechanism applies to DHS associated with Parkinson's disease (PD) and multiple system atrophy (MSA) while the latter applies to DHS associated with amyotrophic lateral sclerosis, myasthenia gravis, polymyositis, and cerebral infarction. DHS is not only caused by neurological conditions; there have been reports of anti-PD drugs, cervical laminoplasty, and radiotherapy causing iatrogenic DHS. A large comparative study on the general population reported the prevalence of DHS in PD patients to be 16.2% [3].

Abnormal posture accompanying PD and related disorders (PDRD), which may include not only DHS but also camptocormia and Pisa syndrome, are often difficult to treat and may interfere with activities of daily living. Patients with severe pain in the extensor muscles may present with acute inflammation. Hemmi et al. reported that steroid pulse therapy had significant effects on DHS caused by myopathy [4]; however, it did not have an effect on DHS caused by other conditions.

Previously, we performed a study on treatment of camptocormia in patients with PD without dorsal muscle weakness. Based on anatomical information, we performed surface electromyography (EMG) assessments of activities in the various truncal muscles using a tilt table to alter the patients' position from a supine position to a standing position. We found that the standing position and the force of gravity resulted in the abnormal firing of the external oblique muscle [5]. In subsequent studies, we injected lidocaine into the external oblique muscle and reported improvement of the camptocormia [6,7]. Based on these findings, we hypothesized that a similar strategy of assessing the target muscle, and lidocaine injection could be used to treat DHS in patients with PDRD.

Temporary improvement of DHS with lidocaine injection into the sternocleidomastoid (SCM) muscle has been reported [2,8]. Based on our knowledge of the evaluation and treatment of camptocormia, we hypothesized that we could obtain good therapeutic effects by maintaining the correct posture with rehabilitation after lidocaine injection. Here, we report the long-term effects of this new strategy.

## 2 Patients and methods

This study was a retrospective analysis of treatment prospectively performed.

### 2.1 Participants

From April 2012 to May 2016, 37 patients were admitted to our hospital for treatment of DHS. The exclusion criteria comprised: improvement with rehabilitation or medication adjustment, obvious neck extensor muscle weakness, limited range of motion due to cervical spine lesions, obvious cognitive impairment, difficulty with periodical evaluation or refusal of lidocaine injection. As there was a possibility that the dropped head would improve with rest and time off work, the patients underwent an observation period of at least 1 week after hospitalization, during which they received rehabilitation.

This clinical research was carried out at National Center Hospital, National Center of Neurology and Psychiatry (NCNP) with the approval of the Ethics Committee of NCNP. The study was conducted according to the principles of the Declaration of Helsinki. All participants provided informed consent.

### 2.2 Needle EMG assessment

We performed needle EMG assessments of the neck muscles with the participants in a sitting position. The default dropped head position was considered as the natural position. The examiner then extended their necks from the natural position to the passively lifted posture and recorded changes in the needle EMG parameters. The evaluated muscles included the scalene muscle, SCM, levator scapulae muscle (LS), splenius capitis muscle (SC), and trapezius muscle (Tz).

### 2.3 Lidocaine injection and neck corset

Patients with DHS had strong neck flexion, which made it difficult for them to wear neck corsets. We injected 2.5 ml of 1% lidocaine into the bilateral scalene muscles (total daily dose was 5 ml). The patient with MSA was injected with twice as much lidocaine per day (total daily dose was 10 ml). The injections were given once a day for 4 or 5 days. The patients were immediately fitted with the neck corset after each injection. We selected an appropriate neck corset for each participant that could hold their neck in the correct position. First, we tried rigid neck collars such as metal frames or VISTA*®* collars. Soft neck collars were used when subjects refused to wear rigid neck collars because of discomfort. The participants were instructed to continue wearing the corsets. They continued rehabilitation (massage, stretching, self-correction in front of the mirror) during the study period.

### 2.4 Assessment

We defined the neck flexion angle as the angle between the horizontal line and the line passing through the ear canal and the external eye angle (O-E line). The horizontal line was set as a reference where downward angles were represented by + while upward angles were represented − degrees. We measured the neck flexion angles at pre-injection (baseline), Day 8, and Day 90.

The two criteria for a diagnosis of DHS were 1) a neck flexion angle of 0° or more and forward bending of the neck, and 2) self-reported obstacles to daily life due to forward bending of the neck. Five neurologists (YM, YM, YH, TK, and YS) independently measured the neck flexion angles. Median values were compared between time points with the Wilcoxon signed-rank test, with *P* < .05 being considered statistically significant. Data were reported as the mean ± standard deviation.

## 3 Results

We enrolled 7 males and 8 females (mean age: 68.9 years, range 56 to 85 years) who met the criteria (Supplemental figure and table). The subjects included 13 patients with PD, 1 patient with MSA, and 1 patient with progressive supranuclear palsy. The mean disease duration of PDRD was 8.0 ± 4.7 years while that of DHS was 1.7 ± 2.7 years. The Hohen and Yahr stage of participants was between 2 and 4. Eleven out of 15 participants were taking non-ergot dopamine-receptor agonists. They had not changed agonists for more than a month, and could not cease this medication because of motor fluctuation.

**Fig. 1:**
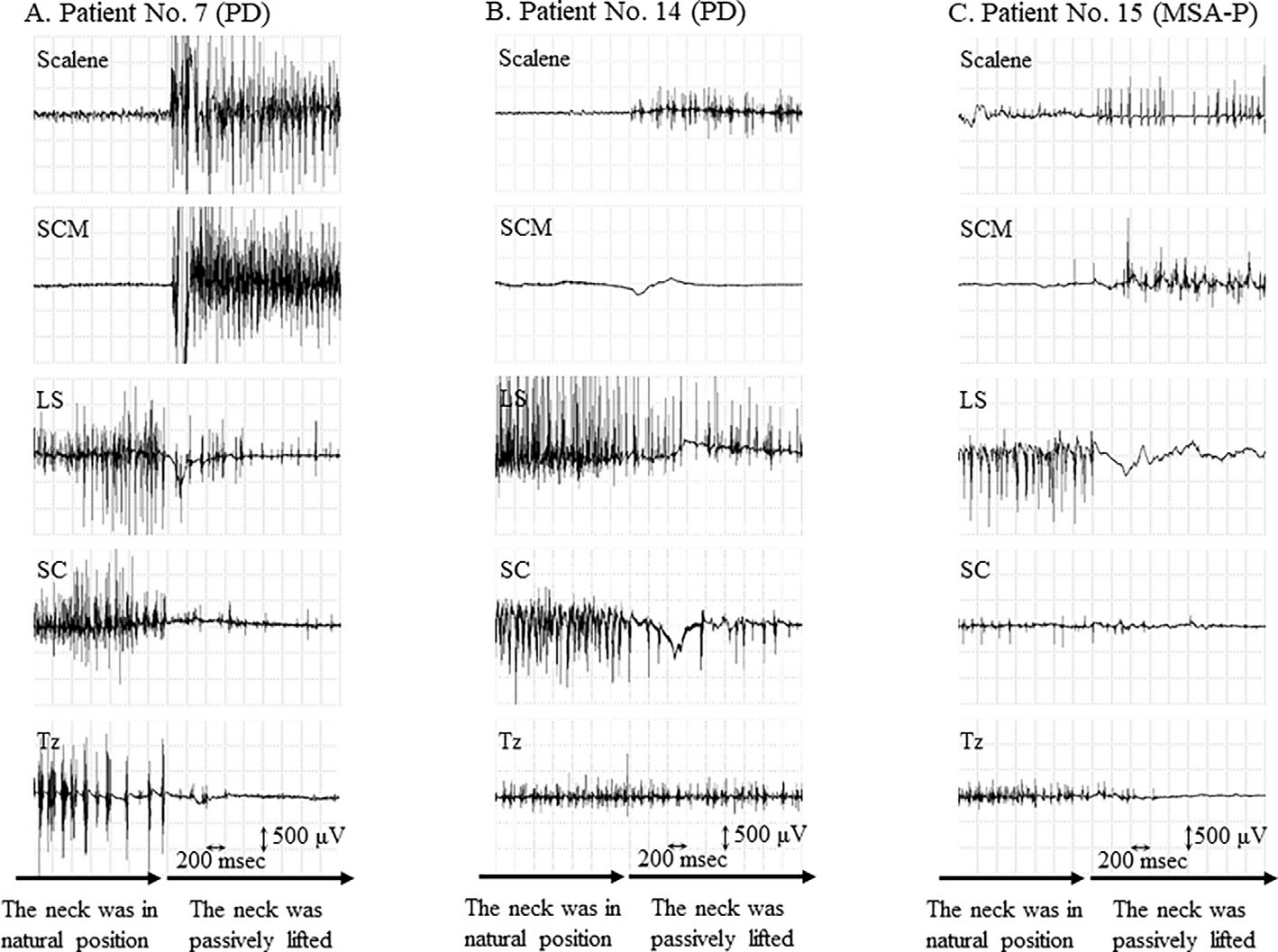
Needle electromyography findings of patients with DHS caused by Parkinson's disease and related disorders PD, Parkinson's disease; MSA-P, multiple system atrophy with predominant Parkinsonian features; SCM, sternocleidomastoid muscle; LS, levator scapulae muscle; Tz, trapezius muscle; SC, splenius capitis muscle.

Findings of needle EMG assessment performed on the 13 patients are shown in Fig. 1. In the natural position, no discharge was observed in the scalene and SCM muscles, while the LS, SC, and Tz muscles showed a sustained discharge. When the examiner corrected the head position of the patient, there was apparent discharge of the scalene and SCM muscles, while that of the LS, SC, and Tz muscles was reduced or disappeared (Fig. 1A,C). These typical findings in the scalene muscles were found in all 13 patients; however, 4 patients did not show the typical findings of the SCM muscle (Fig. 1B).

**Fig. 2:**
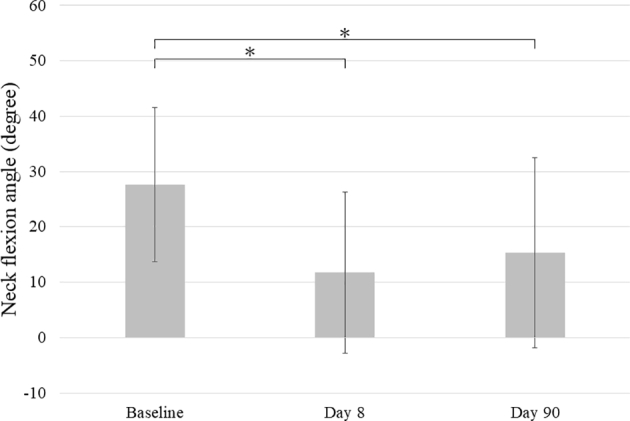
Average flexion angle after lidocaine injection and neck corset wearing Error bars indicate standard deviation. * *P* < .05.

All participants received the combination therapy (lidocaine injection into the scalene muscles and wearing the neck corset). At Day 8, the combination therapy had significantly improved the DHS. The average neck flexion angles at baseline, Day 8, and Day 90 were 27.7° ± 13.9°, 11.7° ± 14.6° (*p* = .0012), and 15.3° ± 17.2° (*p* = .0214), respectively (Fig. 2).

## 4 Discussion

Despite there being previous reports and clinical observations, an effective treatment for DHS experienced by patients with PDRD is yet to be established. In this study, we presented promising results for DHS treatment by combining scalene muscle lidocaine injections and neck corset wearing that have clear clinical implications.

Using the same evaluation and treatment protocol as that previously used in patients with camptocormia [[5], [6], [7]], we found that the causative muscle of DHS is the scalene muscle. Based on surface EMG results, Oyama [8] and Lin [2] suggested that SCM muscle contraction at a normal position triggers neck flexion and that gravitational force maintains the dropped head position. Furthermore, they hypothesized that the LS, Tz, and hindneck muscles are activated as a counterbalance to this gravitational force. We had similar electrophysiological results; however, they did not provide a description of the scalene muscles. Indeed, EMG assessment of the scalene muscles has generally not been previously reported in patients with DHS. This could be due to either the impact of the scalene muscles being downplayed, or the technical difficulties of performing surface EMG assessments of the scalene muscles.

Anatomically, the muscles related to neck flexion are the SCM, scalene, suprahyoid, infrahyoid, longus capitis, and longus cervicis muscles. We focused on the scalene and SCM muscles because the other muscles are either small or deeply located, and hence it is difficult to perform safe and precise injections into them. The scalene muscles originate from the cervical transverse processes and attach to the first and second ribs whereas the SCM muscle is not connected to the cervical spine. We hypothesized that the scalene muscles are more involved in DHS than the SCM muscle in terms of directly bending the cervical spine forward. Indeed, needle EMG assessment of four patients showed no typical changes in the SCM while all the patients showed typical changes in the scalene muscles.

Lidocaine and ethanol injection into the bilateral SCM muscles has been reported to reduce abnormal muscle contraction and to be effective for DHS [[9], [10], [11]]. However, these reports did not investigate the long-term therapeutic effects. Indeed, Oyama et al. reported that the effects were temporary [8]. Previously, we administered lidocaine injections into the bilateral SCM muscles without neck corset wearing as treatment for DHS; however, we did not find any short-term or long-term treatment effects. In addition, the effect of botulinum toxin injections into the SCM muscles is limited [8,11,12]. Fujimoto demonstrated that lidocaine and ethanol preferentially block small gamma-motor nerves while botulinum toxin mainly blocks alpha-motor nerve terminals. Given this difference, lidocaine and ethanol reduce muscle activity without weakening the injected muscles [11]. There have been reports of botulinum toxin injection into the SCM muscle causing dysphagia [8,11,12]. Patients with PDRD often already have clinical or subclinical dysphagia. Thus, lidocaine is safer than botulinum toxin because of its short duration of action and comparative inexpensiveness. Given the demonstrated good therapeutic effect of lidocaine injection, we do not perceive a rationale for using botulinum toxin.

Expanding the movable range using lidocaine injections alleviated pain and discomfort of the jaw and made it possible to wear the neck corset. We speculated that maintaining the head and neck in the correct position for a long time after lidocaine injection is necessary for the treatment of DHS. Without neck muscle tension, the neck tends to bend forward because the center of gravity of the skull lies anterior to the extended line of the cervical spine. We speculate that it is important to continue the neck posture correction even when the subjects are not actively maintaining their head position. In order to prove this hypothesis, it is necessary to compare three groups: using only corset group, lidocaine injection group, corset and lidocaine combination group.

Our study has certain limitations. Firstly, the sample size was too small to verify the effect of the primary disease or the disease duration of DHS on the responses to the intervention. We intend to perform a study with more patients with DHS undergoing combination therapy of lidocaine injections and neck corset wearing and examine factors affecting the therapeutic effect. Secondly, this study was a pilot study and was not randomized. Thirdly, although some of the patients had abnormal needle EMG findings of the SCM muscle, we did not verify the therapeutic effect of combining lidocaine injection into the SCM muscle and neck corset wearing. Fourthly, we could not quantify the optimal wearing period and daily wearing time of the neck corset. Finally, neck MRI imaging was not performed to exclude any associations with isolated neck extensor myopathy. However, the needle EMG did not indicate any findings suspicious of myopathy.

In conclusion, we found that lidocaine injection into the scalene muscles and neck corset wearing resulted in long-term improvement of DHS in patients with PDRD.

The following are the supplementary data related to this article.

**Supplemental figure:**
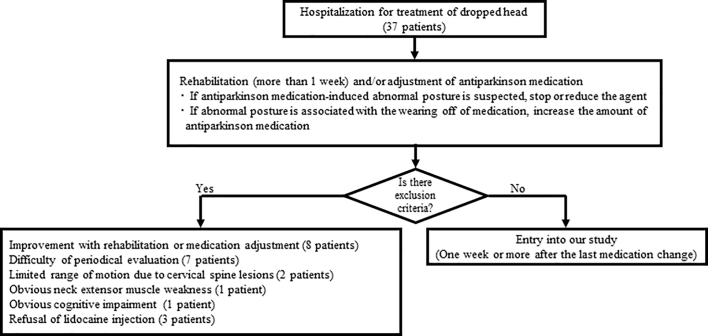
Flow chart of the treatment strategy for patients with dropped head.

**Supplemental table:**
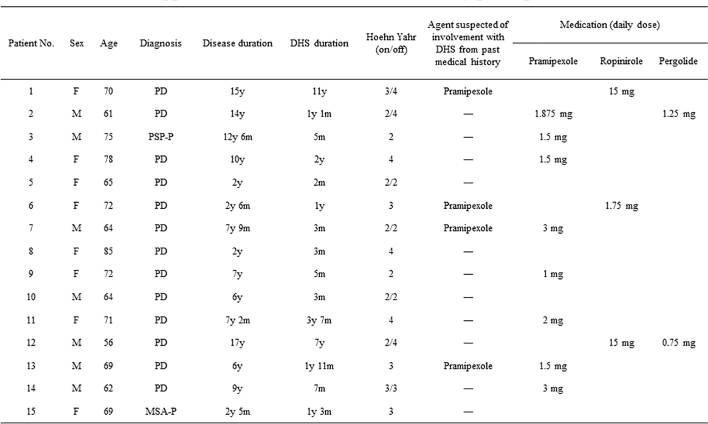
Characteristics of study participants.

## Declaration of competing interest

None.

## References

[1] Lange D.J., Fetell M.R., Lovelace R.E., Rowland L.P. (1986). The floppy head syndrome (abstract). Ann. Neurol..

[2] Lin H., Nagaoka M., Hayashi Y., Yonezawa I. (2013). Pathophysiological analysis of dropped head syndrome caused by various diagnoses —based on surface EMG findings and responses to physiotherapy. Clin. Neurol..

[3] Ando Y., Fujimoto K.I., Ikeda K., Utsumi H., Okuma Y., Oka H., Kamei S., Kurita A., Takahashi K., Nogawa S., Hattori N., Hirata K., Fukui T., Yamazaki K., Yamamoto T., Yoshii F. (2019). Postural abnormality in Parkinson's disease: a large comparative study with general population. Mov. Disord. Clin. Pract..

[4] Hemmi S., Kurokawa K., Izawa N., Kutoku Y., Murakami T., Sunada Y. (2011). Dramatic response of dropped head sign to treatment with steroid in Parkinson's disease: report of three cases. Intern. Med..

[5] Furusawa Y., Hanakawa T., Mukai Y., Aihara Y., Taminato T., Iawata Y., Takei T., Sakamoto T., Murata M. (2015). Mechanism of camptocormia in Parkinson's disease analyzed by tilt table-EMG recording. Parkinsonism Relat. Disord..

[6] Furusawa Y., Mukai Y., Kobayashi Y., Sakamoto T., Murata M. (2012). Role of the external oblique muscle in upper camptocormia for patients with Parkinson's disease. Mov. Disord..

[7] Furusawa Y., Mukai Y., Kawazoe T., Sano T., Nakamura H., Sakamoto C., Iwata Y., Wakita M., Nakata Y., Kamiya K., Kobayashi Y., Sakamoto T., Takiyama Y., Murata M. (2013). Long-term effect of repeated lidocaine injections into the external oblique for upper camptocormia in Parkinson's disease. Parkinsonism Relat. Disord..

[8] Oyama G., Hayashi A., Mizuno Y., Hattori N. (2009). Mechanism and treatment of dropped. Head syndrome associated with parkinsonism. Parkinsonism Relat. Disord..

[9] Yoshiyama Y., Takama J., Hattori T. (1999). The dropped head sign in parkinsonism. J. Neurol. Sci..

[10] Asai H., Udaka F., Kubori T., Matsui H., Oda M., Nishinaka K., Kameyama M. (2005). The efficacy of muscle afferent block for the dropped head of Parkinson's disease. Sogo Rehabilitation.

[11] Fujimoto K. (2006). Dropped head in Parkinson's disease. J. Neurol..

[12] Quinn N.P. (1993). Parkinsonism and dystonia, pseudo-parkinsonism and pseudodystonia. Adv. Neurol..

